# Supplementary material for: HIV-1 RNA in extracellular vesicles is associated with neurocognitive outcomes
Source: Nat Commun. 2024 May 23;15:4391. doi: 10.1038/s41467-024-48644-z (PMC11116485; doi:10.1038/s41467-024-48644-z)
Supplement: Supplementary file 1 — Supplementary Information [file 41467_2024_48644_MOESM1_ESM.pdf]

HIV-1 RNA in Extracellular Vesicles is Associated with Neurocognitive Outcomes

Catherine DeMarino[1], Julia Denniss[1], Maria Cowen[1], Gina Norato[2], Devon K. Dietrich[1], Lisa Henderson[1], Elyse Gollump[3], Joseph Snow[3], Darshan Pandya[1], Bryan Smith[1], Avindra Nath[1]

[1]Section for Infections of the Nervous System, National Institute of Neurological Disorders and Stroke, National Institutes of Health, Bethesda, MD
[2]Office of the Clinical Director, National Institute of Neurological Disorders and Stroke, National Institutes of Health, Bethesda, MD
[3]Office of the Clinical Director, National Institute of Mental Health, National Institutes of Health, Bethesda, MD

Supplementary Table 1. Cross-Section Demographics

Patient	Age*	Sex	Race	Education, years	Duration HIV infection, years	Duration untreated HIV, years	Nadir CD4	CD4 count**	Viral load ***	Drug Regimen	Ever on AZT	Ever on EFV	Ever on "D-drug"
1	59.6	Male	Black	14	12.9	0.2	204	836		Abacavir, Lamivudine, Dolutegravir		Y	
2	56.1	Male	White	12	38.9	15.0	66	560		Darunavir, Ritonavir, Lamivudine			
3	60.8	Female	Black	10	18.5	0.5	6	261		Emtricitabine, Tenofovir AF, Darunavir, Cobicistat, Rilpivirine			
4	59.3	Female	White	14	33.1	8.9	9	1167		Abacavir, Dolutegravir, Lamivudine	Y	Y	Y
5	61.2	Female	Black	9	11.5	5.3	282	1249		Rilpivirine, Tenofovir AF, Emtricitabine			
6	66.1	Male	Black	15	24.1	0.0	4	503		Abacavir, Lamivudine, Efavirenz, DolutegravirORplacebo, MaravirocORplacebo	Y	Y	Y
7	59.5	Male	White	14	27.0	0.0	209	587		Dolutegravir, Rilpivirine	Y	Y	
8	55.2	Female	Black	12	23.2	13.6	230	815		Elvitegravir, Cobicistat, Tenofovir DF, Emtricitabine			
9	61.2	Male	Black	12	24.5	0.8	5	441		Emtricitabine, Tenofovir AF, Raltegravir	Y	Y	Y
10	51.6	Male	Black	8	26.5	22.0	64	499		Abacavir, Lamivudine, Tenofovir DF, Atazanavir, Cobicistat			
11	57.2	Female	Black	12	25.7	10.0	1	913		Elvitegravir, Cobicistat, Tenofovir AF, Emtricitabine	Y		
12	60.4	Female	Black	12	33.1	0.0	199	1255		Efavirenz, Emtricitabine, Tenofovir DF		Y	
13	59.2	Male	Black	11	30.7	14.0	150	984		Darunavir, Cobicistat, Dolutegravir, Rilpivirine			
14	57.2	Male	Black	13	31.8	21.9	46	446		Darunavir, Ritonavir, Etravirine, Raltegravir			
15	65.4	Female	Black	16	35.0	13.0	98	274	172	Emtricitabine, Tenofovir AF, Darunavir, Dolutegravir, Ritonavir	Y		
16	60.0	Male	White	16	35.7	28.0	301	407		Abacavir, Dolutegravir, Lamivudine			
17	58.1	Male	Black	12	12.2	0.0	N/A	808		Emtricitabine, Tenofovir DF, Darunavir, Ritonavir			
18	57.5	Male	White	16	12.9	3.1	182	511		Emtricitabine, Tenofovir DF, Raltegravir			
19	53.2	Female	Black	14	21.2	0.0	174	685		Elvitegravir, Cobicistat, Tenofovir AF, Emtricitabine, Ritonavir	Y	Y	
20	58.4	Male	White	20	5.8	1.0	75	135		Abacavir, Lamivudine, Etravirine			
21	56.0	Male	Black	13	33.6	24.0	24	812		Bictegravir, Tenofovir AF, Emtricitabine			
22	64.3	Female	Black	11	13.1	0.0	420	735		Bictegravir,Tenofovir AF, Emtricitabine		Y	
23	55.5	Male	Black	18	29.2	2.8	7	444		Emtricitabine, Tenofovir DF	Y		
24	56.0	Female	Black	12	19.2	0.0	264	900		Efavirenz, Emtricitabine, Tenofovir DF		Y	
25	59.2	Female	Black	11	20.1	12.2	132	1526		Dolutegravir, Rilpivirine			
26	61.6	Male	White	16	34.4	6.0	50	875		Elvitegravir, Cobicistat, Tenofovir AF, Emtricitabine			
27	61.7	Female	Black	19	29.1	4.3	215	1037		Bictegravir, Tenofovir AF, Emtricitabine		Y	N/A
28	57.4	Female	Other	14	20.3	0.0	377	715		Emtricitabine, Tenofovir AF, Dolutegravir	Y	Y	
29	34.8	Male	White	18	4.3	0.4	650	932		Emtricitabine, Tenofovir DF, Raltegravir			
30	53.9	Female	Black	12	13.8	8.2	676	1787		Bictegravir, Tenofovir AF, Emtricitabine			
31	52.8	Male	White	7	10.5	0.2	0	593		Bictegravir, Tenofovir AF, Emtricitabine		Y	
32	64.2	Male	White	18	20.0	0.0	200	547		Bictegravir, Tenofovir AF, Emtricitabine	Y	Y	
33	49.5	Male	White	17	17.0	3.0	200	546		Emtricitabine, Tenofovir DF, Nevirapine			
34	63.3	Male	White	19	27.2	1.2	160	708		Abacavir, Dolutegravir, Lamivudine		Y	Y
35	59.6	Male	White	14	21.3	5.3	75	444		Elvitegravir, Cobicistat, Tenofovir AF, Emtricitabine, Darunavir	Y		
36	64.3	Female	Black	11	25.9	0.0	80	1189		Bictegravir, Tenofovir AF, Emtricitabine		Y	
37	58.8	Male	Black	16	5.9	0.0	199	216		Efavirenz, Emtricitabine, Tenofovir DF, Abacavir		Y	
38	43.4	Female	White	19	23.2	1.0	12	841		Lopinavir, Ritonavir, Abacavir, Lamivudine			

39	51.7	Female	White	17	27.1	17.3	186	789	Emtricitabine, Tenofovir AF, Atazanavir, Cobicistat	Y	Y	Y
40	62.0	Male	White	20	31.0	0.0	400	556	Fosamprenavir, Abacavir, Ritonavir, Efavirenz		Y	Y
41	54.1	Female	Black	14	19.7	12.5	692	1119	Elvitegravir, Cobicistat,Tenofovir AF, Emtricitabine	Y	Y	
42	60.7	Male	Black	14	30.2	20.0	426	484	Bictegravir, Tenofovir AF, Emtricitabine			
43	46.4	Male	Black	14	27.2	25.7	42	180	Emtricitabine, Tenofovir AF, Etravirine			
44	47.2	Male	White	13	3.2	0.0	465	1062	Abacavir, Dolutegravir, Lamivudine			
45	56.7	Male	Black	14	20.3	0.0	573	848	Bictegravir, Tenofovir AF, Emtricitabine	Y		
46	52.8	Female	Black	8	8.2	0.3	203	802	Rilpivirine,Tenofovir AF, Emtricitabine		Y	
47	49.1	Male	Black	12	2.6	0.0	201	907	Elvitegravir, Cobicistat, Tenofovir DF, Emtricitabine			
48	56.4	Male	White	20	11.4	5.2	48	414	Emtricitabine, Tenofovir AF, Dolutegravir			
49	63.7	Male	White	13	5.9	1.9	64	413	Abacavir, Dolutegravir, Lamivudine			
50	56.5	Female	White	12	7.4	0.1	0	555	Elvitegravir, Cobicistat, Tenofovir AF, Emtricitabine		Y	
51	39.1	Male	Black	14	18.7	0.3	N/A	447	Elvitegravir, Cobicistat, Tenofovir DF, Emtricitabine	Y	Y	
52	48.3	Male	Black	15	12.9	3.0	199	733	Bictegravir, Tenofovir AF, Emtricitabine		Y	
53	61.8	Male	Black	9	25.0	3.0	199	507	Rilpivirine, Tenofovir AF, Emtricitabine, Dolutegravir			
54	57.2	Male	White	16	33.1	8.0	199	675	Bictegravir, Tenofovir AF, Emtricitabine			
55	65.3	Female	Black	16	27.8	4.7	131	592	Emtricitabine, Tenofovir AF, Doravirine	Y	Y	N/A
56	56.2	Male	White	17	18.2	0.1	230	408	Elvitegravir, Cobicistat, Tenofovir AF, Emtricitabine	Y		
57	58.4	Male	White	16	30.3	6.0	30	223	Elvitegravir, Cobicistat, Tenofovir AF, Emtricitabine, Darunavir			
58	57.8	Male	White	18	4.1	0.1	475	631	Bictegravir, Tenofovir AF, Emtricitabine			N/A
59	27.3	Male	White	16	4.3	0.0	657	657	Elvitegravir, Cobicistat, Tenofovir AF, Emtricitabine			
60	64.2	Male	Black	16	36.9	28.0	199	495	Bictegravir, Tenofovir AF, Emtricitabine			
61	79.6	Male	White	18	22.9	0.0	0	915	Bictegravir, Tenofovir AF, Emtricitabine	Y		N/A
62	56.7	Male	White	13	31.5	0.1	70	417	Emtricitabine, Tenofovir AF, Darunavir, Cobicistat, Dolutegravir			
63	55.4	Male	White	14	33.8	3.0	150	492	Abacavir, Dolutegravir, Lamivudine, Tenofovir DF			
64	54.9	Male	White	18	29.0	0.1	199	762	Abacavir, Dolutegravir, Lamivudine	Y		Y
65	53.1	Female	Black	13	28.3	0.4	N/A	446	Rilpivirine, Tenofovir AF, Emtricitabine			
66	64.2	Male	White	14	11.1	2.0	249	256	Abacavir, Dolutegravir, Lamivudine			
67	38.4	Male	White	19	4.9	0.2	199	641	Bictegravir, Tenofovir AF, Emtricitabine		Y	
68	37.1	Male	Black	12	8.2	0.2	N/A	622	Abacavir, Dolutegravir, Lamivudine			
69	50.4	Female	Black	12	21.4	0.0	2	863	Rilpivirine, Tenofovir AF, Emtricitabine			
70	56.1	Female	White	16	32.6	24.1	135	268	Bictagravir, Tenofovir AF, Emtricitabine, Maraviroc			
71	70.4	Male	White	16	32.2	6.3	99	504	Emtricitabine, Tenofovir AF, Dolutegravir	Y		N/A
72	76.4	Female	Black	10	19.4	5.0	20	702	Abacavir, Dolutegravir, Lamivudine	Y		
73	58.2	Male	Black	12	10.5	0.0	201	670	Rilpivirine, Tenofovir AF, Emtricitabine			
74	40.3	Male	Black	18	13.7	10.5	323	924	Bictegravir, Tenofovir AF, Emtricitabine			
75	50.8	Male	Black	11	27.6	5.3	35	970	Abacavir, Dolutegravir, Lamivudine			
76	59.4	Male	Other	8	21.9	10.0	150	892	Emtricitabine, Tenofovir AF, Raltegravir			N/A
77	57.5	Male	White	13	29.0	0.4	400	812	Bictegravir, Tenofovir AF, Emtricitabine			N/A
78	68.6	Male	Black	13	34.8	2.0	213	375	Bictegravir, Tenofovir AF, Emtricitabine		Y	N/A
79	52.2	Female	Black	12	18.1	0.2	35	314	Emtricitabine, Tenofovir AF, Dolutegravir			N/A
80	64.4	Male	White	12	34.0	3.0	495	724	Dolutegravir, Rilpivirine			N/A
81	78.5	Male	White	16	26.8	0.4	277	327	Abacavir, Dolutegravir, Lamivudine	Y		N/A

82	66.1	Female	White	17	31.5	3.0	201	361	Bictegravir, Tenofovir AF, Emtricitabine		N/A
83	62.0	Female	Black	12	18.0	2.0	60	398	Bictegravir, Tenofovir AF, Emtricitabine		N/A
84	54.8	Male	White	16	34.2	3.8	100	595	Abacavir, Dolutegravir, Lamivudine	Y	N/A

*Age at neuropsychological visit
**Measured within 6 months of neuropsychological testing
*** Measured within 6 months of neuropsychological testing, <40 unless otherwise indicated
N/A= data not available; Tenofovir AF = tenofovir alafenamide; Tenofovir DF = tenofovir disoproxil

Supplementary Table 2. Longitudinal Demographics

Patient	Age*	Sex	Race	Education, years	Duration HIV infection, years	Duration untreated HIV, years	Nadir CD4	CD4 count**	Viral Load ***	Drug Regimen	Ever on AZT	Ever on EFV	Ever "D-drug"
2	51.0	Male	White	12	33.8	15.0	66	637		Didanosine, Lamivudine, Ritonavir, Saquinavir			
	53.1				35.8			473		Didanosine, Lamivudine, Ritonavir, Saquinavir			
	56.1				38.9			560		Darunavir, Lamivudine, Ritonavir			
3	57.7	Female	Black	10	15.5	0.5	6	283		Emtricitabine, Rilpivirine, Tenofovir DF			
	59.9				17.6			313		Darunavir, Emtricitabine, Rilpivirine, Tenofovir AF, Cobicistat			
	60.8				18.5			261		Darunavir, Emtricitabine, Rilpivirine, Tenofovir AF, Cobicistat			
4	54.1	Female	White	14	27.9	8.9	9	927		Efavirenz, Emtricitabine, Tenofovir DF	Y	Y	Y
	57.6				31.4			1030		Abacavir, Dolutegravir, Lamivudine			
	59.3				33.1			1167		Abacavir, Dolutegravir, Lamivudine			
5	57.2	Female	Black	9	7.4	5.3	282	894		Emtricitabine, Rilpivirine, Tenofovir DF			
	59.4				9.6			1439		Emtricitabine, Rilpivirine, Tenofovir DF			
	61.2				11.5			1249		Emtricitabine, Rilpivirine, Tenofovir DF			
6	60.9	Male	Black	15	18.9	0.0	4	348		Abacavir, Efavirenz, Lamivudine	Y	Y	Y
	62.0				20.0			418		Abacavir, Efavirenz, Lamivudine			
	64.1				22.1			330		Abacavir, Dolutegravir OR placebo, Efavirenz, Lamivudine, Maraviroc OR placebo			
	66.1				24.1			503		Abacavir, Dolutegravir OR placebo, Efavirenz, Lamivudine, Maraviroc OR placebo			
7	55.4	Male	White	14	22.9	0.0	209	615		Emtricitabine, Tenofovir DF	Y	Y	
	58.6				26.1			678		Dolutegravir, Rilpivirine			
	59.5				27.0			587		Dolutegravir, Rilpivirine			
8	53.1	Female	Black	12	21.1	13.6	230	771		Elvitegravir, Emtricitabine, Tenofovir DF, Cobicistat			
	55.2				23.2			815		Elvitegravir, Emtricitabine, Tenofovir DF, Cobicistat			
	57.2				25.2			694		Elvitegravir, Emtricitabine, Tenofovir DF, Cobicistat			
9	57.2	Male	Black	12	20.5	0.8	5	416		Emtricitabine, Raltegravir, Rilpivirine, Tenofovir DF	Y	Y	Y
	60.1				23.5			479		Emtricitabine, Raltegravir, Rilpivirine, Tenofovir DF			
	61.2				24.5			441		Emtricitabine, Raltegravir, Tenofovir AF			
11	54.0	Female	Black	12	22.5	10.0	1	905		Abacavir, Atazanavir, Lamivudine	Y		
	56.2				24.7			569	27760	Elvitegravir, Emtricitabine, Tenofovir AF, Cobicistat			
	57.2				25.7			913		Elvitegravir, Emtricitabine, Tenofovir AF, Cobicistat			
12	57.2	Female	Black	12	30.0	0.0	<200	1300		Efavirenz, Emtricitabine, Tenofovir DF		Y	
	59.3				32.1			1141		Efavirenz, Emtricitabine, Tenofovir DF			
	60.4				33.1			1255		Efavirenz, Emtricitabine, Tenofovir DF			
13	55.1	Male	Black	11	26.6	14.0	150	623		Darunavir, Dolutegravir, Rilpivirine, Cobicistat			
	56.3				27.7			683		Darunavir, Dolutegravir, Rilpivirine, Cobicistat			
	59.2				30.7			815		Darunavir, Dolutegravir, Rilpivirine, Cobicistat			
14	51.9	Male	Black	13	26.5	21.9	46	213		Darunavir, Etravirine, Raltegravir, Ritonavir			
	55.2				29.7			290		Darunavir, Etravirine, Raltegravir, Ritonavir			
	57.2				31.8			446		Darunavir, Etravirine, Raltegravir, Ritonavir			
15	60.1	Female	Black	16	29.7	13.0	98	490		Lamivudine, Nelfinavir, Zidovudine	Y		
	63.4				32.9			264	67	Darunavir, Dolutegravir, Emtricitabine, Ritonavir, Tenofovir AF			
	65.4				35.0			274	172	Darunavir, Dolutegravir, Emtricitabine, Ritonavir, Tenofovir AF			
16	55.1	Male	White	16	30.8	28.0	301	333		Abacavir, Dolutegravir, Lamivudine			
	57.4				33.1			420		Abacavir, Dolutegravir, Lamivudine			
	60.0				35.7			407		Abacavir, Dolutegravir, Lamivudine			
18	53.5	Male	White	16	8.8	3.1	182	500		Emtricitabine, Raltegravir, Tenofovir DF			
	55.4				10.8			640		Emtricitabine, Raltegravir, Tenofovir DF			
	57.5				12.9			511		Emtricitabine, Raltegravir, Tenofovir DF			
19	48.5	Female	Black	14	16.4	0.0	174	1081		Abacavir, Lamivudine, Ritonavir	Y	Y	
	50.7				18.6			1375		Abacavir, Darunavir, Lamivudine, Ritonavir			
	53.2				21.2			685		Elvitegravir, Emtricitabine, Ritonavir, Tenofovir AF, Cobicistat			
21	51.9	Male	Black	13	29.5	24.0	24	678		Darunavir, Dolutegravir, Emtricitabine, Ritonavir, Tenofovir DF			

	54.1				31.7			822	Abacavir, Darunavir, Dolutegravir, Lamivudine, Cobicistat			
	56.0				33.6			812	Bictegravir, Emtricitabine, Tenofovir AF			
22	57.9	Female	Black	11	6.8	0.0	420	493	Efavirenz, Emtricitabine, Tenofovir DF		Y	
	61.2				10.0			685	Efavirenz, Emtricitabine, Tenofovir DF			
	64.3				13.1			591	Bictegravir, Emtricitabine, Tenofovir DF			
25	57.3	Female	Black	11	18.1	12.2	132	1493	Abacavir, Dolutegravir, Lamivudine			
	59.2				20.1			1526	Dolutegravir, Rilpivirine			
	61.4				22.3			1631	Dolutegravir, Rilpivirine			
85	57.3	Male	Black	8	5.2	0.2	300	517	Efavirenz, Emtricitabine, Tenofovir DF		Y	
	58.7				6.6			596	Efavirenz, Emtricitabine, Tenofovir DF			
	59.6				7.5			521	Efavirenz, Emtricitabine, Tenofovir DF			
26	58.5	Male	White	16	31.3	6.0	50	687	Elvitegravir, Emtricitabine, Tenofovir DF, Cobicistat			
	59.7				32.5			722	Elvitegravir, Emtricitabine, Tenofovir DF, Cobicistat			
	61.6				34.4			875	Elvitegravir, Emtricitabine, Tenofovir DF, Cobicistat			
27	56.6	Female	Black	19	24.1	4.3	215	1113	Atazanavir, Emtricitabine, Ritonavir, Tenofovir DF		Y	Y
	58.8				26.2			1027	Atazanavir, Emtricitabine, Ritonavir, Tenofovir DF			
	61.7				29.1			1037	Bictegravir, Emtricitabine, Tenofovir AF			
30	48.5	Female	Black	12	8.4	8.2	676	1143	Elvitegravir, Emtricitabine, Tenofovir AF, Cobicistat			
	51.7				11.6			1666	Bictegravir, Emtricitabine, Tenofovir AF			
	53.9				13.8			1787	Bictegravir, Emtricitabine, Tenofovir AF			
31	47.4	Male	White	7	5.1	0.2	0	383	Elvitegravir, Emtricitabine, Tenofovir DF, Cobicistat		Y	
	48.8				6.6			471	Elvitegravir, Emtricitabine, Tenofovir DF, Cobicistat			
	50.5				8.2			463	Bictegravir, Emtricitabine, Tenofovir AF			
	52.8				10.5			593	Bictegravir, Emtricitabine, Tenofovir AF			
32	59.0	Male	White	18	14.7	0.0	200	646	Emtricitabine, Rilpivirine, Tenofovir AF	Y	Y	
	61.0				16.7			722	Bictegravir, Emtricitabine, Tenofovir AF			
	64.2				20.0			547	Bictegravir, Emtricitabine, Tenofovir AF			
33	47.9	Male	White	17	15.4	3.0	200	656	Emtricitabine, Nevirapine, Tenofovir DF			
	49.5				17.0			546	Emtricitabine, Nevirapine, Tenofovir DF			
	51.2				18.6			262	Bictegravir, Emtricitabine, Tenofovir AF			
35	58.6	Male	White	14	20.3	5.3	75	640	Darunavir, Elvitegravir, Emtricitabine, Tenofovir AF, Cobicistat	Y		
	59.6				21.3			444	Darunavir, Elvitegravir, Emtricitabine, Tenofovir AF, Cobicistat			
	60.6				22.2			693	Darunavir, Elvitegravir, Emtricitabine, Tenofovir AF, Cobicistat			
36	59.2	Female	Black	11	20.7	0.0	80	1492	Efavirenz, Emtricitabine, Tenofovir DF		Y	
	62.2				23.8			1264	Efavirenz, Emtricitabine, Tenofovir DF			
	64.3				25.9			1189	Bictegravir, Emtricitabine, Tenofovir AF			
39	46.6	Female	White	17	22.0	17.3	186	581	Atazanavir, Emtricitabine, Ritonavir, Tenofovir DF,	Y	Y	Y
	49.7				25.1			818	Atazanavir, Emtricitabine, Tenofovir AF, Cobicistat			
	51.7				27.1			789	Atazanavir, Emtricitabine, Tenofovir AF, Cobicistat			
40	58.8	Male	White	20	27.7	0.0	400	464	Abacavir, Efavirenz, Fosamprenavir, Ritonavir		Y	Y
	61.0				29.9			508	Abacavir, Efavirenz, Fosamprenavir, Ritonavir			
	62.0				31.0			556	Abacavir, Efavirenz, Fosamprenavir, Ritonavir			
41	51.1	Female	Black	14	16.7	12.5	692	1147	Efavirenz, Emtricitabine, Tenofovir DF	Y	Y	
	53.1				18.6			1191	Elvitegravir, Emtricitabine, Tenofovir AF, Cobicistat			
	54.1				19.7			1119	Elvitegravir, Emtricitabine, Tenofovir AF, Cobicistat			
42	56.5	Male	Black	14	26.1	20.0	426	950	Elvitegravir, Emtricitabine, Tenofovir AF, Cobicistat			
	57.7				27.2			677	Elvitegravir, Emtricitabine, Tenofovir AF, Cobicistat			
	59.7				29.2			450	Bictegravir, Emtricitabine, Tenofovir AF			
	60.7				30.2			484	Bictegravir, Emtricitabine, Tenofovir AF			
86	58.2	Male	Black	12	23.1	0.0	123	200	Darunavir, Emtricitabine, Tenofovir DF, Cobicistat		Y	Y
	59.3				24.2			126	Darunavir, Emtricitabine, Tenofovir DF, Cobicistat			
45	54.4	Male	Black	14	17.9	0.0	573	964	Elvitegravir, Emtricitabine, Tenofovir AF, Cobicistat	Y		
	55.6				19.2			897	Elvitegravir, Emtricitabine, Tenofovir AF, Cobicistat			
	56.7				20.3			848	Bictegravir, Emtricitabine, Tenofovir AF			
46	48.8	Female	Black	8	4.1	0.3	203	769	Emtricitabine, Rilpivirine, Tenofovir AF		Y	

	50.9				6.3			886	Emtricitabine, Rilpivirine, Tenofovir AF		
	52.8				8.2			802	Emtricitabine, Rilpivirine, Tenofovir AF		
	54.5				9.4			554	Dolutegravir, Emtricitabine, Tenofovir AF		
48	55.5	Male	White	20	10.4	5.2	48	756	Dolutegravir, Emtricitabine, Tenofovir AF		
	56.4				11.4			414	Dolutegravir, Emtricitabine, Tenofovir AF		
	61.6				3.9			292	Abacavir, Dolutegravir, Lamivudine		
49	62.7	Male	White	13	5.0	1.9	64	362	Abacavir, Dolutegravir, Lamivudine		
	63.7				5.9			413	Abacavir, Dolutegravir, Lamivudine		
	46.0				10.6			805	Abacavir, Efavirenz, Lamivudine		
52	47.1	Male	Black	15	11.7	3.0	<200	829	Abacavir, Efavirenz, Lamivudine		Y
	48.3				12.9			733	Bictegravir, Emtricitabine, Tenofovir AF		
	59.6				22.7	3.0		568	Dolutegravir, Emtricitabine, Rilpivirine, Tenofovir AF		
53	61.8	Male	Black	9	25.0		<200	507	Dolutegravir, Emtricitabine, Rilpivirine, Tenofovir AF		
	64.0				27.1			691	Dolutegravir, Emtricitabine, Rilpivirine, Tenofovir AF		
	55.1				31.0			634	Etravirine, Maraviroc, Raltegravir		
54	56.1	Male	White	16	32.0	8.0	<200	634	Bictegravir, Emtricitabine, Maraviroc, Tenofovir AF		
	57.2				33.1			675	Bictegravir, Emtricitabine, Tenofovir AF		
	55.0				17.0			534	Elvitegravir, Emtricitabine, Tenofovir AF, Cobicistat		
56	56.2	Male	White	17	18.2	0.1	230	408	Elvitegravir, Emtricitabine, Tenofovir AF, Cobicistat	Y	
	57.9				19.9			467	Bictegravir, Emtricitabine, Tenofovir AF		
	60.8				33.5			595	Emtricitabine, Raltegravir, Tenofovir AF		
60	62.0	Male	Black	16	34.7	28.0	<200	555	Bictegravir, Emtricitabine, Tenofovir AF		
	64.2				36.9			495	Bictegravir, Emtricitabine, Tenofovir AF		
	49.2				26.0			748	Abacavir, Dolutegravir, Lamivudine		
75	50.8	Male	Black	11	27.6	5.3	35	970	Abacavir, Dolutegravir, Lamivudine		
	52.0				28.8			752	Cabotegravir, Rilpivirine		

*Age at neuropsychological visit
**Measured within 6 months of neuropsychological testing
*** Measured within 6 months of neuropsychological testing, <40 at all timepoints unless otherwise indicated
Tenofovir AF = tenofovir alafenamide; Tenofovir DF = tenofovir disoproxil

a

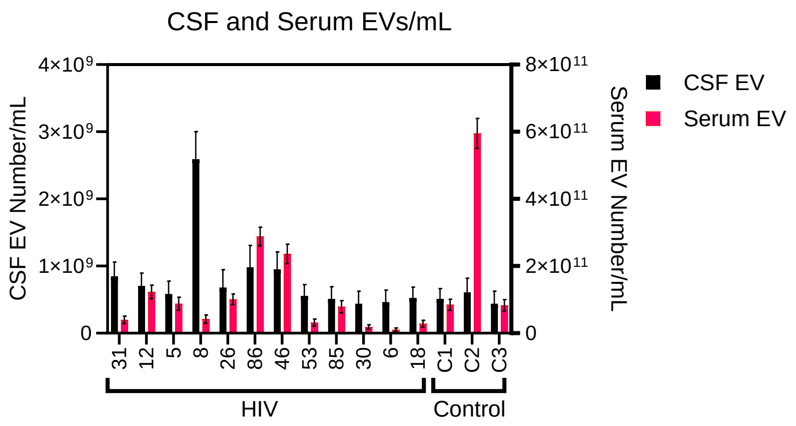

b

	Mean CSF EVs	Mean Serum EVs
Mean CSF EVs	-	0.952
Mean Serum EVs	0.952	-
CSF TAR	0.906	0.500
CSF Long LTR	0.427	0.401
Serum TAR	0.645	0.584
Serum Long LTR	0.710	0.057
GDS	1	0.914
T-Score	0.824	0.430
Verbal Domain Score	0.438	0.642
SIP Domain Score	0.768	0.915
Executive Functioning Domain Score	0.350	0.017
Working Memory Domain Score	0.871	0.979
Learning Domain Score	0.366	0.601
Memory Domain Score	0.547	0.894
Motor Domain Score	0.821	0.352

Supplementary Figure 1. **Extracellular vesicle quantity does not correlate with viral RNAs. a** CSF and serum EVs were quantitated using a microfluidic cartridge nanoparticle analyzer. **b** Pearson correlation was used to investigate the relationship between EV quantities (mean CSF EVs; n=12 CSF samples or mean serum EVs n=12 serum samples), viral RNA copy numbers, and neuropsychological data.

Supplementary Table 3. Summary of cross-sectional correlation coefficients and (p-values).

	CSF Readthrough	CSF TAR	CSF Long LTR	CSF Pol	CSF Tat-Rev	CSF Nef	CSF PolyA	Serum Readthrough	Serum TAR	Serum Long LTR	Serum Pol	Serum Tat-Rev	Serum Nef	Serum PolyA	GDS	Overall T Score	Verbal Fluency	SIP	Executive Functioning	Attention/Working Memory	Learning	Memory	Psychomotor
**CSF Readthrough**	1 (0)	0.07 (0.777)	**0.468 (0)**	**0.166 (0.025)**	-0.002 (0.186)	**0.255 (0.015)**	-0.136 (0.36)	-0.229 (0.273)	0.125 (0.411)	0.155 (0.298)	-0.042 (0.859)	-0.166 (0.295)	-0.044 (0.974)	0.068 (0.586)	0.108 (0.271)	-0.141 (0.445)	0.025 (0.685)	-0.217 (0.197)	-0.08 (0.33)	-0.118 (0.422)	0.071 (0.419)	-0.062 (0.994)	**-0.299 (0.022)**
**CSF TAR**	0.07 (0.777)	1 (0)	-0.027 (0.18)	0.112 (0.997)	**0.312 (0.016)**	0.126 (0.87)	0.052 (0.988)	**-0.31 (0.028)**	-0.073 (0.71)	-0.028 (0.795)	-0.206 (0.22)	-0.063 (0.587)	-0.031 (0.657)	-0.209 (0.413)	0.023 (0.719)	-0.077 (0.503)	0.057 (0.838)	-0.072 (0.383)	-0.011 (0.873)	0.114 (0.903)	0.025 (0.578)	-0.036 (0.897)	-0.018 (0.799)
**CSF Long LTR**	**0.468 (0)**	-0.027 (0.18)	1 (0)	**0.078 (0.03)**	-0.068 (0.65)	**0.222 (0.017)**	0.059 (0.77)	-0.028 (0.581)	0.128 (0.208)	0.168 (0.227)	-0.218 (0.201)	-0.125 (0.239)	-0.184 (0.36)	0.092 (0.953)	0.215 (0.057)	**-0.388 (0.011)**	-0.083 (0.334)	-0.314 (0.093)	**-0.317 (0.001)**	**-0.422 (0.048)**	-0.255 (0.11)	-0.26 (0.109)	-0.156 (0.208)
**CSF Pol**	**0.166 (0.025)**	0.112 (0.997)	**0.078 (0.03)**	1 (0)	0.028 (0.647)	**0.238 (0.005)**	**-0.267 (0.019)**	0.181 (0.352)	-0.024 (0.858)	-0.06 (0.565)	-0.016 (0.779)	0.068 (0.481)	0.045 (0.479)	0.08 (0.689)	0.081 (0.349)	-0.019 (0.905)	0.092 (0.703)	0.014 (0.841)	0.065 (0.768)	-0.041 (0.882)	-0.004 (0.937)	-0.04 (0.94)	0.136 (0.38)
**CSF Tat-Rev**	-0.002 (0.186)	**0.312 (0.016)**	-0.068 (0.65)	0.028 (0.647)	1 (0)	-0.2 (0.188)	**-0.259 (0.034)**	0.098 (0.918)	-0.025 (0.986)	0.211 (0.14)	-0.121 (0.345)	0.133 (0.379)	0.033 (0.716)	-0.104 (0.169)	0.009 (0.676)	-0.022 (0.632)	0.225 (0.212)	-0.071 (0.307)	-0.002 (0.886)	-0.016 (0.904)	-0.111 (0.399)	-0.096 (0.406)	0.042 (0.899)
**CSF Nef**	**0.255 (0.015)**	0.126 (0.87)	**0.222 (0.017)**	**0.238 (0.005)**	-0.2 (0.188)	1 (0)	0.193 (0.237)	-0.212 (0.103)	-0.074 (0.58)	**-0.297 (0.026)**	-0.163 (0.247)	-0.173 (0.276)	-0.198 (0.289)	0.013 (0.612)	0.101 (0.532)	-0.121 (0.734)	-0.043 (0.985)	-0.197 (0.13)	0.077 (0.341)	-0.075 (0.763)	0 (0.572)	0.001 (0.605)	-0.126 (0.231)
**CSF PolyA**	-0.136 (0.36)	0.052 (0.988)	0.059 (0.77)	**-0.267 (0.019)**	**-0.259 (0.034)**	0.193 (0.237)	1 (0)	-0.153 (0.258)	0.058 (0.885)	-0.051 (0.946)	-0.101 (0.384)	-0.148 (0.283)	-0.073 (0.387)	-0.032 (0.844)	-0.082 (0.819)	0.056 (0.585)	-0.004 (0.789)	0.098 (0.425)	-0.03 (0.895)	0.01 (0.99)	-0.096 (0.419)	-0.078 (0.543)	0.002 (0.97)
**Serum Readthrough**	-0.229 (0.273)	**-0.31 (0.028)**	-0.028 (0.581)	0.181 (0.352)	0.098 (0.918)	-0.212 (0.103)	-0.153 (0.258)	1 (0)	0.086 (0.732)	**0.266 (0.012)**	0.178 (0.5)	0.039 (0.635)	0.099 (0.686)	0.023 (0.588)	0.124 (0.418)	-0.082 (0.878)	-0.065 (0.845)	0.045 (0.51)	-0.105 (0.923)	-0.027 (0.78)	-0.28 (0.125)	-0.223 (0.214)	0.067 (0.823)
**Serum TAR**	0.125 (0.411)	-0.073 (0.71)	0.128 (0.208)	-0.024 (0.858)	-0.025 (0.986)	-0.074 (0.58)	0.058 (0.885)	0.086 (0.732)	1 (0)	0.173 (0.63)	0.192 (0.091)	**0.233 (0.055)**	**0.332 (0.002)**	-0.069 (0.451)	0.195 (0.088)	-0.103 (0.19)	0.149 (0.363)	0.1 (0.168)	-0.018 (0.636)	0.042 (0.368)	-0.198 (0.347)	-0.168 (0.265)	-0.201 (0.183)
**Serum Long LTR**	0.155 (0.298)	-0.028 (0.795)	0.168 (0.227)	-0.06 (0.565)	0.211 (0.14)	**-0.297 (0.026)**	-0.051 (0.946)	**0.266 (0.012)**	0.173 (0.63)	1 (0)	0.15 (0.459)	0.011 (0.723)	0.272 (0.109)	-0.077 (0.611)	0.154 (0.146)	-0.244 (0.079)	-0.083 (0.761)	-0.107 (0.436)	-0.185 (0.346)	-0.13 (0.289)	-0.209 (0.054)	-0.229 (0.078)	-0.223 (0.098)
**Serum Pol**	-0.042 (0.859)	-0.206 (0.22)	-0.218 (0.201)	-0.016 (0.779)	-0.121 (0.345)	-0.163 (0.247)	-0.101 (0.384)	0.178 (0.5)	0.192 (0.091)	0.15 (0.459)	1 (0)	0.108 (0.191)	**0.37 (0.001)**	**0.242 (0.048)**	0.1 (0.606)	-0.131 (0.271)	-0.201 (0.172)	-0.069 (0.663)	-0.027 (0.631)	0.072 (0.469)	-0.105 (0.927)	-0.069 (0.889)	-0.105 (0.414)
**Serum Tat-Rev**	-0.166 (0.295)	-0.063 (0.587)	-0.125 (0.239)	0.068 (0.481)	0.133 (0.379)	-0.173 (0.276)	-0.148 (0.283)	0.039 (0.635)	0.233 (0.055)	0.011 (0.723)	0.108 (0.191)	1 (0)	**0.255 (0.04)**	0.056 (0.486)	-0.122 (0.273)	0.135 (0.315)	0.044 (0.623)	0.179 (0.164)	-0.074 (0.919)	-0.105 (0.519)	0.126 (0.217)	0.18 (0.06)	0.205 (0.162)
**Serum Nef**	-0.044 (0.974)	-0.031 (0.657)	-0.184 (0.36)	0.045 (0.479)	0.033 (0.716)	-0.198 (0.289)	-0.073 (0.387)	0.099 (0.686)	**0.332 (0.002)**	0.272 (0.109)	**0.37 (0.001)**	**0.255 (0.04)**	1 (0)	0.039 (0.805)	0.003 (0.914)	0.068 (0.497)	0.161 (0.208)	0.117 (0.315)	0.079 (0.912)	0.135 (0.269)	0.054 (0.524)	0.012 (0.728)	0.045 (0.823)
**Serum PolyA**	0.068 (0.586)	-0.209 (0.413)	0.092 (0.953)	0.08 (0.689)	-0.104 (0.169)	0.013 (0.612)	-0.032 (0.844)	0.023 (0.588)	-0.069 (0.451)	-0.077 (0.611)	**0.242 (0.048)**	0.056 (0.486)	0.039 (0.805)	1 (0)	0.086 (0.792)	-0.132 (0.347)	-0.202 (0.211)	-0.038 (0.811)	-0.174 (0.259)	-0.145 (0.326)	0.005 (0.66)	-0.062 (0.906)	-0.108 (0.456)
**GDS**	0.108 (0.271)	0.023 (0.719)	0.215 (0.057)	0.081 (0.349)	0.009 (0.676)	0.101 (0.532)	-0.082 (0.819)	0.124 (0.418)	0.195 (0.088)	0.154 (0.146)	0.1 (0.606)	-0.122 (0.273)	0.003 (0.914)	0.086 (0.792)	**1 (0)**	**-0.878 (0)**	**-0.368 (0.007)**	**-0.601 (0)**	**-0.719 (0)**	**-0.522 (0)**	**-0.762 (0)**	**-0.75 (0)**	**-0.402 (0.004)**
**Overall T Score**	-0.141 (0.445)	-0.077 (0.503)	**-0.388 (0.011)**	-0.019 (0.905)	-0.022 (0.632)	-0.121 (0.734)	0.056 (0.585)	-0.082 (0.878)	-0.103 (0.19)	-0.244 (0.079)	-0.131 (0.271)	0.135 (0.315)	0.068 (0.497)	-0.132 (0.347)	**-0.878 (0)**	**1 (0)**	**0.583 (0)**	**0.747 (0)**	**0.707 (0)**	**0.588 (0)**	**0.771 (0)**	**0.78 (0)**	**0.406 (0.001)**
**Verbal Fluency**	0.025 (0.685)	0.057 (0.838)	-0.083 (0.334)	0.092 (0.703)	0.225 (0.212)	-0.043 (0.985)	-0.004 (0.789)	-0.065 (0.845)	0.149 (0.363)	-0.083 (0.761)	-0.201 (0.172)	0.044 (0.623)	0.161 (0.208)	-0.202 (0.211)	**-0.368 (0.007)**	**0.583 (0)**	**1 (0)**	**0.362 (0)**	**0.373 (0.003)**	**0.304 (0.007)**	**0.264 (0.012)**	**0.248 (0.022)**	0.197 (0.086)
**SIP**	-0.217 (0.197)	-0.072 (0.383)	-0.314 (0.093)	0.014 (0.841)	-0.071 (0.307)	-0.197 (0.13)	0.098 (0.425)	0.045 (0.51)	0.1 (0.168)	-0.107 (0.436)	-0.069 (0.663)	0.179 (0.164)	0.117 (0.315)	-0.038 (0.811)	**-0.601 (0)**	**0.747 (0)**	**0.362 (0)**	**1 (0)**	**0.487 (0.001)**	**0.406 (0)**	**0.49 (0)**	**0.558 (0)**	**0.384 (0)**

**Executive Functioning**	-0.08<br>(0.33)	-0.011<br>(0.873)	**-0.317<br>(0.001)**	0.065<br>(0.768)	-0.002<br>(0.886)	0.077<br>(0.341)	-0.03<br>(0.895)	-0.105<br>(0.923)	-0.018<br>(0.636)	-0.185<br>(0.346)	-0.027<br>(0.631)	-0.074<br>(0.919)	0.079<br>(0.912)	-0.174<br>(0.259)	**-0.719<br>(0)**	**0.707<br>(0)**	**0.373<br>(0.003)**	**0.487<br>(0.001)**	**1<br>(0)**	**0.578<br>(0)**	**0.536<br>(0)**	**0.528<br>(0)**	**0.361<br>(0.003)**
**Attention/Working Memory**	-0.118<br>(0.422)	0.114<br>(0.903)	**-0.422<br>(0.048)**	-0.041<br>(0.882)	-0.016<br>(0.904)	-0.075<br>(0.763)	0.01<br>(0.99)	-0.027<br>(0.78)	0.042<br>(0.368)	-0.13<br>(0.289)	0.072<br>(0.469)	-0.105<br>(0.519)	0.135<br>(0.269)	-0.145<br>(0.326)	**-0.522<br>(0)**	**0.588<br>(0)**	**0.304<br>(0.007)**	**0.406<br>(0)**	**0.578<br>(0)**	**1<br>(0)**	**0.371<br>(0)**	**0.425<br>(0)**	0.175<br>(0.062)
**Learning**	0.071<br>(0.419)	0.025<br>(0.578)	-0.255<br>(0.11)	-0.004<br>(0.937)	-0.111<br>(0.399)	0<br>(0.572)	-0.096<br>(0.419)	-0.28<br>(0.125)	-0.198<br>(0.347)	-0.209<br>(0.054)	-0.105<br>(0.927)	0.126<br>(0.217)	0.054<br>(0.524)	0.005<br>(0.66)	**-0.762<br>(0)**	**0.771<br>(0)**	**0.264<br>(0.012)**	**0.49<br>(0)**	**0.536<br>(0)**	**0.371<br>(0)**	**1<br>(0)**	**0.898<br>(0)**	**0.226<br>(0.01)**
**Memory**	-0.062<br>(0.994)	-0.036<br>(0.897)	-0.26<br>(0.109)	-0.04<br>(0.94)	-0.096<br>(0.406)	0.001<br>(0.605)	-0.078<br>(0.543)	-0.223<br>(0.214)	-0.168<br>(0.265)	-0.229<br>(0.078)	-0.069<br>(0.889)	0.18<br>(0.06)	0.012<br>(0.728)	-0.062<br>(0.906)	**-0.75<br>(0)**	**0.78<br>(0)**	**0.248<br>(0.022)**	**0.558<br>(0)**	**0.528<br>(0)**	**0.425<br>(0)**	**0.898<br>(0)**	**1<br>(0)**	**0.266<br>(0.004)**
**Psychomotor**	**-0.299<br>(0.022)**	-0.018<br>(0.799)	-0.156<br>(0.208)	0.136<br>(0.38)	0.042<br>(0.899)	-0.126<br>(0.231)	0.002<br>(0.97)	0.067<br>(0.823)	-0.201<br>(0.183)	-0.223<br>(0.098)	-0.105<br>(0.414)	0.205<br>(0.162)	0.045<br>(0.823)	-0.108<br>(0.456)	**-0.402<br>(0.004)**	**0.406<br>(0.001)**	0.197<br>(0.086)	**0.384<br>(0)**	**0.361<br>(0.003)**	0.175<br>(0.062)	**0.226<br>(0.01)**	**0.266<br>(0.004)**	**1<br>(0)**

SIP=Speed of Information Processing

Supplementary Table 4. Summary of longitudinal correlation coefficients and (p-values).

	CSF Long LTR	Serum Long LTR	CSF TAR	Serum TAR	GDS	Overall T Score	Verbal Fluency	Speed of Information Processing	Executive Functioning	Attention/Working Memory	Learning	Memory	Psychomotor
**CSF Long LTR**	1(0)	0.203(0.192)	0.037(0.813)	0.156(0.316)	-0.179(0.252)	-0.08(0.607)	-0.213(0.17)	-0.099(0.525)	**-0.332(0.03)**	0.028(0.856)	0.116(0.458)	0.122(0.436)	0.046(0.768)
**Serum Long LTR**	0.203(0.192)	1(0)	-0.063(0.69)	0.019(0.903)	-0.272(0.078)	-0.041(0.795)	-0.155(0.32)	0.051(0.742)	-0.122(0.433)	0.292(0.058)	0.113(0.471)	0.013(0.932)	-0.299(0.052)
**CSF TAR**	0.037(0.813)	-0.063(0.69)	1(0)	0.118(0.448)	0.205(0.187)	-0.201(0.196)	0.058(0.713)	-0.101(0.52)	0.033(0.834)	-0.097(0.533)	-0.165(0.288)	-0.225(0.146)	-0.198(0.202)
**Serum TAR**	0.156(0.316)	0.019(0.903)	0.118(0.448)	1(0)	**0.309(0.044)**	0.074(0.636)	-0.072(0.646)	0.156(0.317)	-0.089(0.569)	0.099(0.525)	-0.057(0.714)	-0.032(0.838)	0.211(0.174)
**GDS**	-0.179(0.252)	-0.272(0.078)	0.205(0.187)	**0.309(0.044)**	1(0)	**-0.424(0.005)**	0.052(0.74)	-0.131(0.403)	-0.061(0.695)	-0.207(0.184)	**-0.519(0)**	**-0.615(0)**	-0.061(0.699)
**Overall T Score**	-0.08(0.607)	-0.041(0.795)	-0.201(0.196)	0.074(0.636)	**-0.424(0.005)**	1(0)	**0.422(0.005)**	**0.723(0)**	0.048(0.758)	**0.311(0.043)**	**0.65(0)**	**0.672(0)**	**0.542(0)**
**Verbal Fluency**	-0.213(0.17)	-0.155(0.32)	0.058(0.713)	-0.072(0.646)	0.052(0.74)	**0.422(0.005)**	1(0)	0.247(0.11)	-0.045(0.776)	0.015(0.926)	0.016(0.917)	0.161(0.303)	0.271(0.079)
**Speed of Information Processing**	-0.099(0.525)	0.051(0.742)	-0.101(0.52)	0.156(0.317)	-0.131(0.403)	**0.723(0)**	0.247(0.11)	1(0)	-0.071(0.65)	0.19(0.221)	**0.317(0.039)**	0.244(0.115)	**0.307(0.046)**
**Executive Functioning**	**-0.332(0.03)**	-0.122(0.433)	0.033(0.834)	-0.089(0.569)	-0.061(0.695)	0.048(0.758)	-0.045(0.776)	-0.071(0.65)	1(0)	-0.058(0.71)	-0.059(0.707)	-0.154(0.321)	-0.163(0.294)
**Attention/Working Memory**	0.028(0.856)	0.292(0.058)	-0.097(0.533)	0.099(0.525)	-0.207(0.184)	**0.311(0.043)**	0.015(0.926)	0.19(0.221)	-0.058(0.71)	1(0)	0.108(0.488)	0.194(0.212)	0.07(0.654)
**Learning**	0.116(0.458)	0.113(0.471)	-0.165(0.288)	-0.057(0.714)	**-0.519(0)**	**0.65(0)**	0.016(0.917)	**0.317(0.039)**	-0.059(0.707)	0.108(0.488)	1(0)	**0.633(0)**	0.294(0.056)
**Memory**	0.122(0.436)	0.013(0.932)	-0.225(0.146)	-0.032(0.838)	**-0.615(0)**	**0.672(0)**	0.161(0.303)	0.244(0.115)	-0.154(0.321)	0.194(0.212)	**0.633(0)**	1(0)	**0.441(0.003)**
**Psychomotor**	0.046(0.768)	-0.299(0.052)	-0.198(0.202)	0.211(0.174)	-0.061(0.699)	**0.542(0)**	0.271(0.079)	**0.307(0.046)**	-0.163(0.294)	0.07(0.654)	0.294(0.056)	**0.441(0.003)**	1(0)

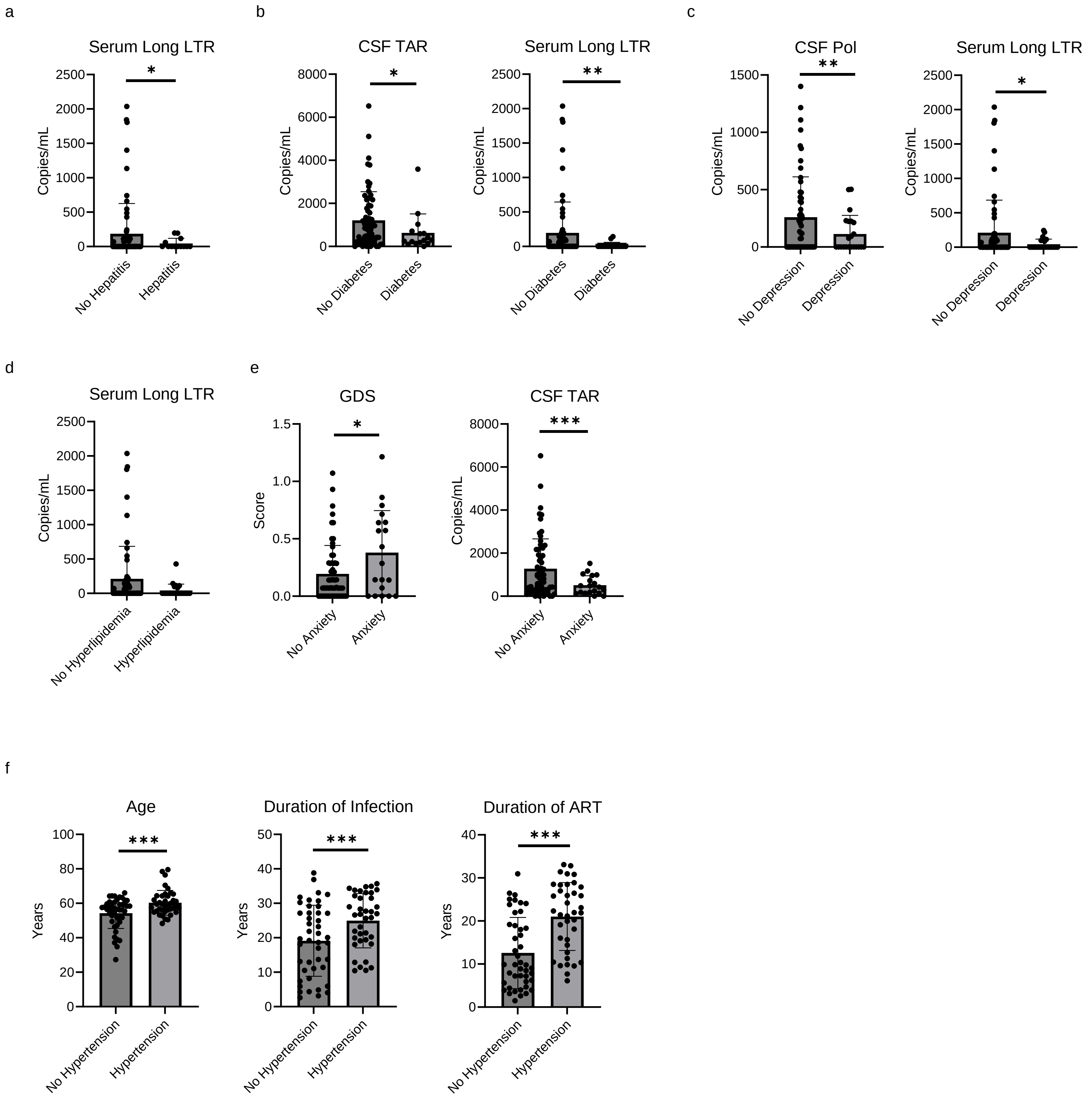

Supplementary Figure 2. **Effect of comorbidities on viral transcription.** Common comorbidities in the tested cohort (n=86) include history of **a** chronic hepatitis C (13/86), **b** type II diabetes (16/86), **c** depression (26/86), **d** hyperlipidemia (25/86), **e** anxiety (19/86), **f** hypertension (39/86). Participants were binned by presence or absence of comorbidity and a student's *t*-test was used to assess for significant variation between groups for all variables. Bars represent mean +SD. Significant findings are shown. *p<0.05, **p<0.01, **p<0.001.

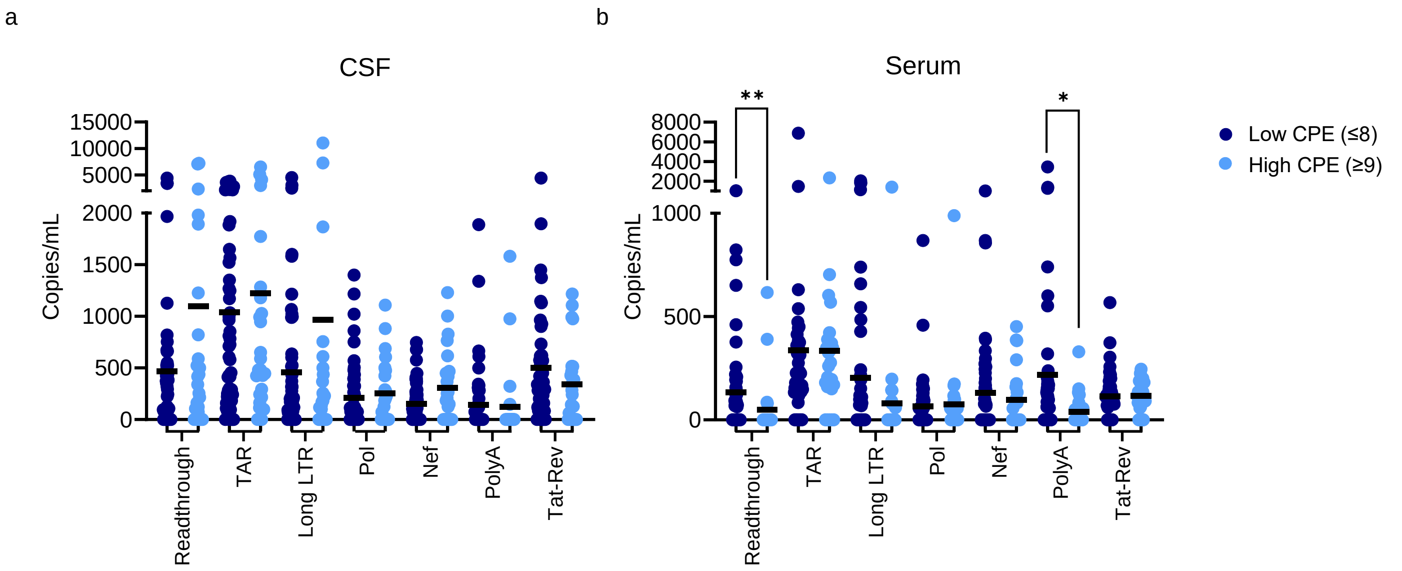

Supplementary Figure 3. **Effect of CNS penetrance of antiretrovirals on viral transcription.** Antiretroviral regimens were scored according to CNS penetration effectiveness (CPE) scores and each individual was binned into low CPE (sum of CPE scores for individual drugs was less than or equal to 8; n=57) or high CPE (sum of CPE scores of individual drugs was greater than or equal to 9, n=27) for CSF **a** and serum **b**. Scores were assigned values as previously published[19-21] with the addition of bictegravir (CPE=3) and doravirine (CPE=4) from written communication with Dr. Scott Letendre (14 December 2023). Bars represent mean of each population. A Mann Whitney test was used to assess data for significant variation between groups. * p=0.03, ** p=0.008

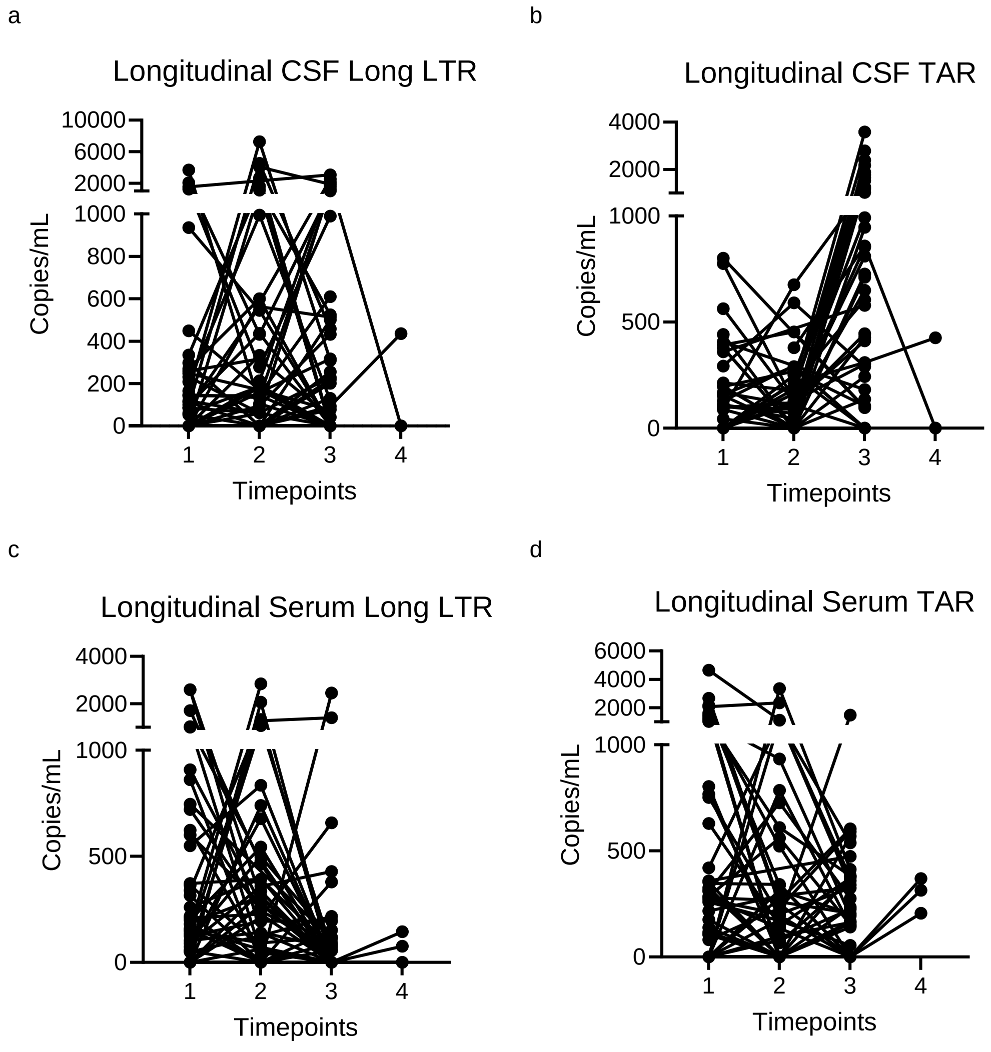

Supplementary Figure 4. **Individual viral transcript trajectories.** Longitudinal copy numbers for each individual (n=43) over test time point for **a** CSF TAR, **b** CSF Long LTR, **c** Serum TAR, and **d** Serum Long LTR. Intervals between timepoints are between 1 and 3 years (Refer to Supplementary Table 2).

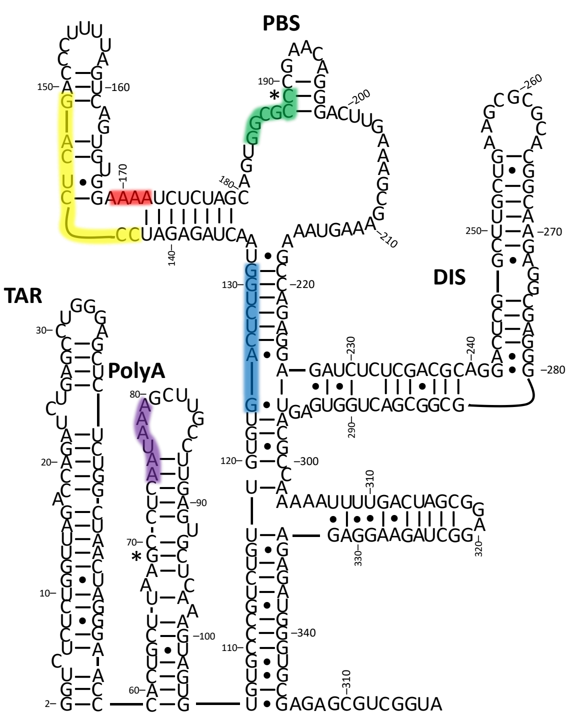

Supplementary Figure 5. **HIV LTR Diagram**. Nucleotides 2 through 317 of the HIV LTR are shown in their 2D structure. The primary components of the LTR are highlighted, the polyA site (purple), primary activation site (blue), tRNA stabilizing motif (yellow), anticodon binding motif (red), and the portion of the primer binding site included in the sequenced amplicon (green). The asterisks indicate the nucleotide sequence amplified by the primers.

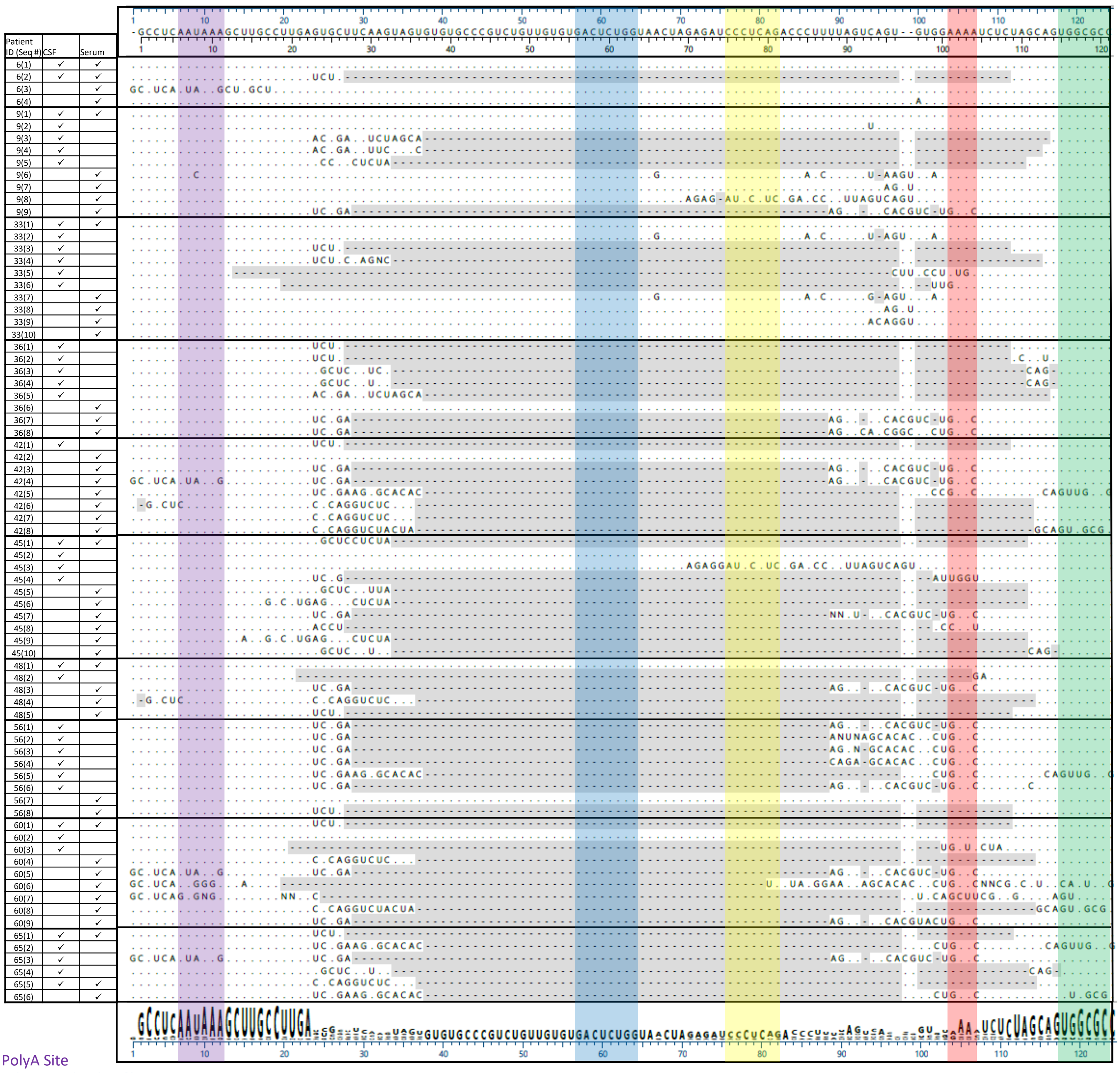

PolyA Site
Primer Activation Site
tRNA Stabilizing Motif
Anticodon Binding Motif
Primer Binding Site

Supplementary Figure 6. **Long LTR amplicon sequence alignment.** The Long LTR sequences for each of the 10 individuals (CSF and serum) were aligned to the HIV reference genome (HXB2, shown on the top). The distribution of the sequence is shown on the left side of the figure indicating if the sequence was found in the CSF, serum or both. The sequence logo is shown on the bottom. The grey regions indicate deleted sequence. The important motifs within the sequences amplicon are highlighted, the polyA site (purple), primary activation site (blue), tRNA stabilizing motif (yellow), anticodon binding motif (red), and the portion of the primer binding site included in the sequenced amplicon (green) and correspond to the highlight motifs depicted in Supplementary Data Figure 5.

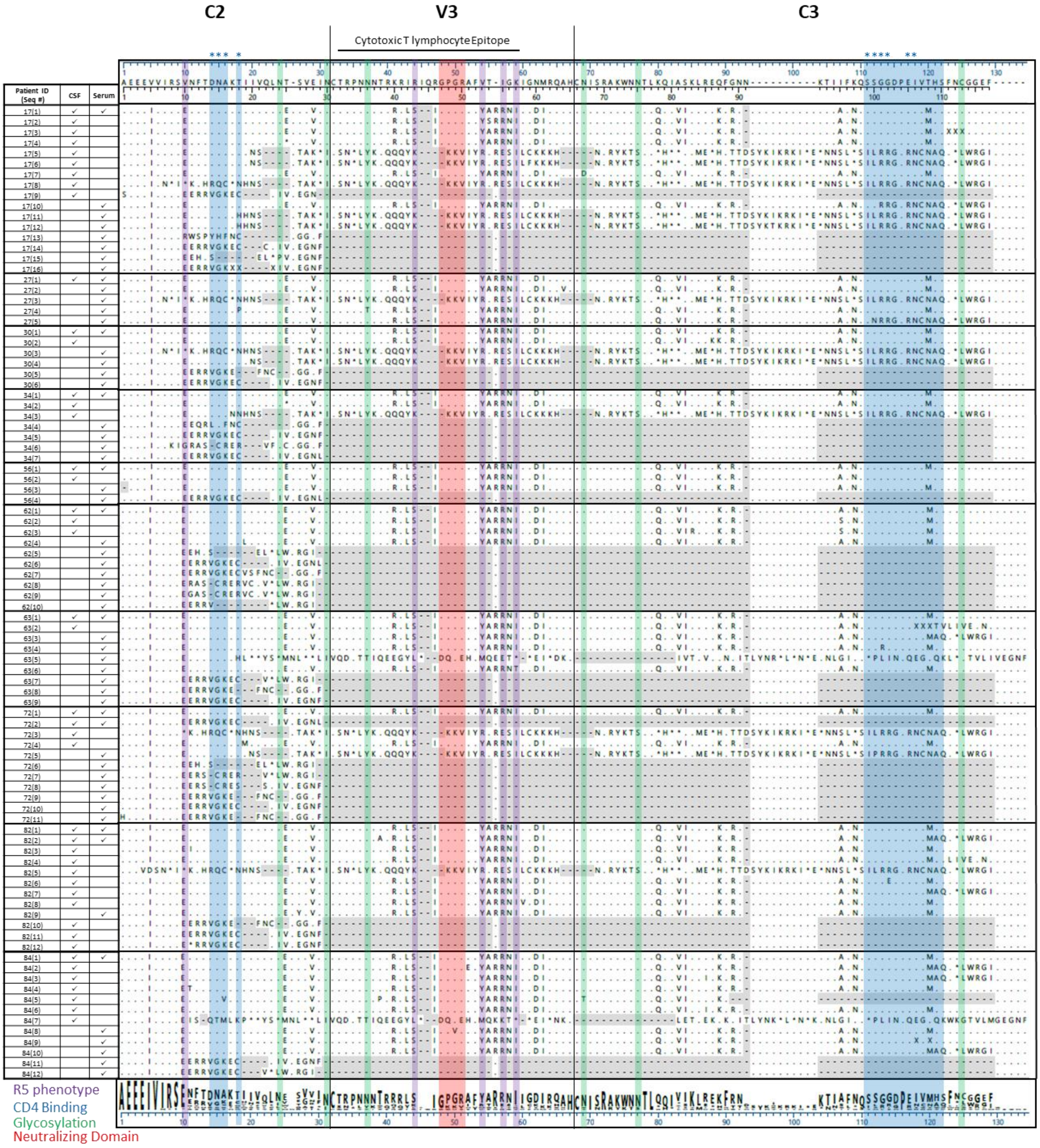

Supplementary Figure 7. **HIV envelope amino acid sequence alignment. The** Long LTR sequences for each of the 10 individuals (CSF and serum) were aligned to the HIV reference genome (HXB2, shown on the top). The distribution of the sequence is shown on the left side of the figure indicating if the sequence was found in the CSF, serum or both. The sequence logo is shown on the bottom. The important motifs within the sequences amplicon are highlighted, amino acids critical for the R5 phenotype (purple), CD4 binding (blue), glycosylation (green), and the neutralizing domain (red).

Supplementary Table 4. Primers and Probes

Gene	Primer	Purpose
Readthrough	F- gccctcagatgctrcatataa<br>R- agagtcacacaacagacgg<br>Probe- tgcctgtactgggtctctctggttag	ddPCR
TAR	F- gtctctctggttagaccag<br>R- tgggttccctagytagcc<br>Probe- agcctgggagctc	ddPCR
Long LTR	F- gcctcaataaagcttgccttga<br>R- gggcgccactgctagaga<br>Probe- ccagagtcacacaacagacgggcaca	ddPCR, Sequencing
Pol	F- gcactttaaattttcccattagtccta<br>R- caaatttctactaatgcttttattttttc<br>Probe- aagccaggaatggatggcc	ddPCR
Nef	F- ggtgggagcagyatctcgaga<br>R- tgtaagtcattggtcttaaaggtacctgagg<br>Probe- ccaggcacaakcagcatt	ddPCR
PolyA	F- gccctcagatgctrcatataa<br>R- ttttttttttttttttttttttttgaag<br>Probe- tgcctgtactgggtctctctggttag	ddPCR
Tat-Rev	F- cttaggcatctcctatggcaggaa<br>R- ggatctgtctctgtctctctctccacc<br>Probe- acccgacaggcc	ddPCR
Envelope	F- gcagaagaagagatagtaattagatc<br>R- aaaattcccctccacaattaaa	Sequencing
