# Supplementary material for: HIV-1 RNA in extracellular vesicles is associated with neurocognitive outcomes
Source: Nat Commun. 2024 May 23;15:4391. doi: 10.1038/s41467-024-48644-z (PMC11116485; doi:10.1038/s41467-024-48644-z)
Supplement: Supplementary file 3 — Reporting Summary [file 41467_2024_48644_MOESM3_ESM.pdf]

nature portfolio

Corresponding author(s): Avi Nath

Last updated by author(s): Mar 30, 2024

Reporting Summary

Nature Portfolio wishes to improve the reproducibility of the work that we publish. This form provides structure for consistency and transparency in reporting. For further information on Nature Portfolio policies, see our Editorial Policies and the Editorial Policy Checklist.

Statistics

For all statistical analyses, confirm that the following items are present in the figure legend, table legend, main text, or Methods section.

n/a	Confirmed	
☐	☒	The exact sample size (*n*) for each experimental group/condition, given as a discrete number and unit of measurement
☐	☒	A statement on whether measurements were taken from distinct samples or whether the same sample was measured repeatedly
☐	☒	The statistical test(s) used AND whether they are one- or two-sided<br>*Only common tests should be described solely by name; describe more complex techniques in the Methods section.*
☐	☒	A description of all covariates tested
☐	☒	A description of any assumptions or corrections, such as tests of normality and adjustment for multiple comparisons
☐	☒	A full description of the statistical parameters including central tendency (e.g. means) or other basic estimates (e.g. regression coefficient) AND variation (e.g. standard deviation) or associated estimates of uncertainty (e.g. confidence intervals)
☐	☒	For null hypothesis testing, the test statistic (e.g. *F*, *t*, *r*) with confidence intervals, effect sizes, degrees of freedom and *P* value noted<br>*Give P values as exact values whenever suitable.*
☒	☐	For Bayesian analysis, information on the choice of priors and Markov chain Monte Carlo settings
☒	☐	For hierarchical and complex designs, identification of the appropriate level for tests and full reporting of outcomes
☐	☒	Estimates of effect sizes (e.g. Cohen's *d*, Pearson's *r*), indicating how they were calculated

Our web collection on statistics for biologists contains articles on many of the points above.

Software and code

Policy information about availability of computer code

Data collection	Digital Droplet PCR was collected and analyzed in Quantasoft 1.7.4. DNA and amino acid sequences were aligned and visualized using Clustal Omega in DNAStar Lasergene MegAlign Pro 17.
Data analysis	Analyses were conducted R version 4.2.0. and GraphPad Prism 10.1.0

For manuscripts utilizing custom algorithms or software that are central to the research but not yet described in published literature, software must be made available to editors and reviewers. We strongly encourage code deposition in a community repository (e.g. GitHub). See the Nature Portfolio guidelines for submitting code & software for further information.

Data

Policy information about availability of data

All manuscripts must include a data availability statement. This statement should provide the following information, where applicable:

- Accession codes, unique identifiers, or web links for publicly available datasets
- A description of any restrictions on data availability
- For clinical datasets or third party data, please ensure that the statement adheres to our policy

The sequence data generated in this study has been deposited on NINDS' supplementary data website - https:// data.ninds.nih.gov. The HIV HXB2 reference genome is accession number K03455 M38432. Source data are provided with this paper.

Research involving human participants, their data, or biological material

Policy information about studies with human participants or human data. See also policy information about sex, gender (identity/presentation), and sexual orientation and race, ethnicity and racism.

Reporting on sex and gender	Self reported sex was included as a covariate in data analysis and was not found to have a significant impact.
Reporting on race, ethnicity, or other socially relevant groupings	Race was self reported under the CDC revised race standards which identifies five categories for data on race; American Indian or Alaska native, Asian, Black or African American, Native Hawaiian or Other Pacific Islander, and White. race was included as a covariate in statistical analysis and was not found to have a significant impact.

Population characteristics

INCLUSION CRITERIA:
All Subjects (HIV-infected and HIV-negative Controls):

While different individual HIV neurocognitive studies have specific selection criteria, especially related to HIV viral load and antiretroviral therapy, inclusion criteria for this overarching protocol will be flexible in order to identify the broadest base of potential enrollees possible.

Men and women, 18 years of age and older
Ability to sign informed consent by the subject
At least seventh grade educational level and ability to speak, read, and understand English. Education level will be assessed by subject self-report. Because many of the neuropsychological subtests were validated using United States norms, subjects must be native English speakers or if foreign-born, demonstrate ability to understand the English language at the time of protocol consent and neuropsychological testing.
Consent to store blood and tissue
Willing to participate in this study for 10 years
HIV-infected Only:

HIV-1 infection, as documented by OraQuick rapid test using venipuncture whole blood, or fingerstick whole blood; or with HIV-1/HIV-2 Multispot rapid test and Western Blot as determined by NIH Clinical Pathology Laboratory or Leidos Biomedical Research. Monitoring Laboratory.
Outside primary medical doctor who provides care
Plasma HIV-RNA <50 copies/mm3 or BLD for greater than one year. Patients who experience transitory episodes of an HIV viral load > 50 copies/mm3 preceded and followed by plasma viremia < 50 copies/mm3 may be included.
At least one year of continuous ART
HIV-negative Controls Only:

1.HIV-antibody negative

EXCLUSION CRITERIA:

Illness or other condition that, in the opinion of the PI, may interfere with study participation at the time of enrollment, including, but not limited to those listed below:

CNS infections: this includes but is not limited to Varicella zoster virus (VZV) encephalitis, CNS lymphoma and toxoplasmosis. Subjects who have recovered from effectively treated CNS infections may be considered once they resume baseline daily activities.
Non-CNS opportunistic infections: subjects who recovered from or are completing treatment for non-CNS opportunistic infections (OIs) (e.g., Pneumocystis pneumonia, Candida esophagitis, or pulmonary TB) can be enrolled if they have returned to self-reported baseline activity and functional level.
Conditions other than HAND associated with cognitive impairment or dementia such as Alzheimer s, Parkinson s disease, head injury with loss of consciousness >30 minutes, untreated sleep apnea with day-time sleepiness, or seizure disorders. Subjects with a history of seizure disorder with no seizure activity that are on a stable, non-sedating anti-seizure regimen for >6 months may be enrolled.
Concurrent severe, unstable psychiatric illness that, in the opinion of the investigators, may interfere with study participation and/or data interpretation. Subjects on psychotropic anxiolytic, attention deficit-hyperactivity disorder (ADHD), and other psychiatric medications may be included if clinically stable for >6 months.
Concurrent substance abuse that, in the opinion of the investigators may interfere with study participation and/or data interpretation. Active substance abuse includes illegal drug use and/or excessive narcotic or alcohol use as determined by the investigator. Urine drug screen will be performed on all subjects. Use of nicotine containing products will not be an exclusion criterion.
Contraindication to MRI/ MRS scanning, including pacemakers or other implanted electrical devices, brain stimulators, some types of dental implants, aneurysm clips (metal clips on the wall of a large artery), metallic prostheses (including metal pins and rods, heart valves, and cochlear implants), implanted delivery pump, or shrapnel fragments. Patients requiring a low dose oral benzodiazepine for mild to moderate claustrophobia will be allowed to participate. Pregnancy testing will be performed in enrolled participants of childbearing potential 48 hours prior to any MRI.
Medications: narcotics, psychiatric, and anti-seizure medications will not be allowed except under certain conditions as noted above. Corticosteroids may be permitted for subjects on stable short-term therapy without CNS disease (i.e., resolving Pneumocystis pneumonia). Participants must be willing not to take the following medications within 48 hours of neuropsychological testing : sedating antihistamines such as diphenhydramine, zolpidem and other drugs identified by the study team that are associated with altered alertness or impaired memory.
Inability to refrain from use of anticoagulant/antiplatelet medication, such as dipyridamole (Persantine), clopidogrel (Plavix), dabigatran (Pradaxa), or warfarin (Coumadin) for at least 72 hours prior to invasive procedures (lumbar puncture [LP], lumbar

drain). Aspirin does not need to be held.
Prior or planned/anticipated exposure to radiation due to clinical care or participation in other research protocols, which would exceed the recommended acceptable annual limit of radiation exposure once accounting for the requirements of the current study.
Pregnant or Lactating females are excluded due to exposure to the radioactive compound for PET/CT scans, which may be excreted in the breast milk and could be potentially harmful to breast-fed infants. There is also exposure to radiation from the CT part of the PET/CT scan and the lumbar puncture if done under fluoroscopy. Women of childbearing potential must have a negative serum or urine pregnancy 48 hours prior to any radiation exposure.

Recruitment	Recruitment was done via advertisement on local buses, mailing flyers, coenrolment from other NIH protocols, advertisement on clinicaltrials.gov, NIH clinical center website. The demographics of the recruited patient population resembles that of the infected population in this area. The study required that the paients be able to take the neuropsychological testing in English. Hence the recruited population was biased towards an English speaking population.
Ethics oversight	All research complies with all relevant ethical regulations and has been approved by the NIH Institutional Review Board (IRB). Informed consent was obtained from all individuals. Financial compensation is provided to participants as per NIH IRB recommendations. IRB number 13N0149

Note that full information on the approval of the study protocol must also be provided in the manuscript.

Field-specific reporting

Please select the one below that is the best fit for your research. If you are not sure, read the appropriate sections before making your selection.

☒ Life sciences ☐ Behavioural & social sciences ☐ Ecological, evolutionary & environmental sciences

For a reference copy of the document with all sections, see nature.com/documents/nr-reporting-summary-flat.pdf

Life sciences study design

All studies must disclose on these points even when the disclosure is negative.

Sample size	The patient population comes from a larger observational clinical trial (NCT01875588) which is a prospective study that aims to recruit over 300 HIV+ individuals. Upon study initiation the estimated proportion of individuals within the cohort with cognitive impairment was estimated at 40-50%. However, results from the study have shown that the rate is approximately 20-25%. Therefore, the study aimed to enroll over 300 individuals to give a 95% confidence limit. For this investigation, we used 84 individuals on whom we had CSF available at multiple times points. The analytical goals were primarily descriptive in nature and the results are not taken as confirmatory findings.
Data exclusions	One individual was excluded from the study because they were found to have a congenital malformation of the ventricles on MRI.
Replication	The digital droplet assay has been thoroughly optimized by those who developed it. Reproducibility of the assay in our lab was validated using serial dilutions of the 835 HIV cell line which contains two copies of HIV. CSF or serum from each participant was analyzed in technical triplicate.
Randomization	The study examines the levels of HIV transcripts in relation to clinical outcomes. The data was analyzed unadjusted and adjusted for covariates including: sex, age, duration of ARV treatment, duration of HIV infection, nadir CD4, and viral load.
Blinding	The digital droplet PCR assay utilized in this study is designed to detect HIV transcripts therefore all individuals included in the analysis were HIV-infected individuals. The selection of CSF/serum samples was determined by availability of the material and researchers were blinded to cognitive scores until time of analysis.

Reporting for specific materials, systems and methods

We require information from authors about some types of materials, experimental systems and methods used in many studies. Here, indicate whether each material, system or method listed is relevant to your study. If you are not sure if a list item applies to your research, read the appropriate section before selecting a response.

Materials & experimental systems

n/a	Involved in the study	
☒	☐	Antibodies
☐	☒	Eukaryotic cell lines
☒	☐	Palaeontology and archaeology
☒	☐	Animals and other organisms
☐	☒	Clinical data
☒	☐	Dual use research of concern
☒	☐	Plants

Methods

n/a	Involved in the study	
☒	☐	ChIP-seq
☒	☐	Flow cytometry
☒	☐	MRI-based neuroimaging

Eukaryotic cell lines

Policy information about cell lines and Sex and Gender in Research

Cell line source(s)	J1.1 (HIV-1 lymphadenopathy-associated virus (LAV)-Infected Jurkat E6 cells (J1.1), ARP-1340 obtained through the NIH HIV Reagent Program, Division of AIDS, NIAID, NIH, contributed by Dr. Thomas Folks)
Authentication	Confirmed to be p24 positive by ELISA and to express viral transcripts by qPCR.
Mycoplasma contamination	The J1.1 cell line used tested negative for mycoplasma contamination.
Commonly misidentified lines (See ICLAC register)	n/a

Clinical data

Policy information about clinical studies

All manuscripts should comply with the ICMJE guidelines for publication of clinical research and a completed CONSORT checklist must be included with all submissions.

Clinical trial registration	NCT01875588
Study protocol	Full description of the protcol can be found on clinicaltrials.gov
Data collection	The natural history of neurocognitive impairment in human immunodeficiency virus (HIV)-infected individuals remains poorly understood. While the advent of highly active antiretroviral therapy (HAART) has led to a decreased incidence of the most severe form of HIV associated neurocognitive disorders (HAND), HIV-associated dementia, it does not appear to have impacted overall prevalence of HAND. Existing evidence suggests that the central nervous system (CNS) could be an important reservoir for HIV regardless of cumulative time on treatment. This 20 year multi-institute natural history protocol will identify approximately 500 HIV-infected individuals and 250 healthy volunteers for enrollment in multiple HAND studies at the National Institutes of Health (NIH). Subjects will undergo a screening and evaluation assessment, which will include blood and urine collection, neuropsychological testing, Client Diagnostic Questionnaire (CDQ), and brain magnetic resonance imaging (MRI) with optional lumbar puncture and ophthalmology exam to repeat yearly for up to ten years.
Outcomes	*Describe how you pre-defined primary and secondary outcome measures and how you assessed these measures.*

Plants

Seed stocks	*Report on the source of all seed stocks or other plant material used. If applicable, state the seed stock centre and catalogue number. If plant specimens were collected from the field, describe the collection location, date and sampling procedures.*
Novel plant genotypes	*Describe the methods by which all novel plant genotypes were produced. This includes those generated by transgenic approaches, gene editing, chemical/radiation-based mutagenesis and hybridization. For transgenic lines, describe the transformation method, the number of independent lines analyzed and the generation upon which experiments were performed. For gene-edited lines, describe the editor used, the endogenous sequence targeted for editing, the targeting guide RNA sequence (if applicable) and how the editor was applied.*
Authentication	*Describe any authentication procedures for each seed stock used or novel genotype generated. Describe any experiments used to assess the effect of a mutation and, where applicable, how potential secondary effects (e.g. second site T-DNA insertions, mosiacism, off-target gene editing) were examined.*
